# Ethnobotanical study of the wild edible and healthy functional plant resources of the Gelao people in northern Guizhou, China

**DOI:** 10.1186/s13002-022-00572-2

**Published:** 2022-12-19

**Authors:** Jian Xie, Fusong Liu, Xiaohuan Jia, Yongxia Zhao, Xiaoqi Liu, Mingxia Luo, Yuqi He, Sha Liu, Faming Wu

**Affiliations:** 1 School of Preclinical Medicine, Zunyi Medical University, Zunyi, 563000 China; 2 School of Pharmacy, Zunyi Medical University, Zunyi, 563000 China; 3 Guizhou Medical and Health Industry Research Institute, Zunyi Medical University, Zunyi, 563000 China; 4 State Key Laboratory of Functions and Applications of Medicinal Plants, Guizhou Medical University, Guiyang, 550025 China

**Keywords:** Ethnobotany, Gelao people, Wild plants, Medicinal plants, National heritage

## Abstract

**Introduction:**

The Gelao people are a unique minority in Southwest China with a unique culture for the utilization of edible plants, including a large number of medicinal plants. They believe that at least 61 species are edible and have medicinal value. Ethnobotany research can reveal the local knowledge of the Gelao people regarding the traditional use of plants and the relationship between this minority and their living environment to help retain and pass on this traditional knowledge forever.

**Methods:**

Edible wild plants and their applied ethnic knowledge were investigated in three counties in northern Guizhou. Gelao residents were the main informants, and literature search, village interviews, participatory observation and quantitative ethnobotany evaluation were used.

**Results:**

A total of 151 species of wild plants in 67 families are collected and eaten by Gelao residents, among which 61 species were considered to have medicinal value, accounting for 40.4% of the total, and 43 were listed in the Chinese Pharmacopoeia. There were 57 plant species with fruits as their edible parts, which are consumed as snacks, followed by 54 species whose young seedlings and leaves are the edible parts, most of which are consumed cold or stir-fried. Other edible parts included roots or rhizomes (bulbs), flowers, whole plants, seeds, fruiting bodies and stems. There were two consumption modes: raw and cooked. Raw foods were mainly consumed as snacks, which mainly comprise fruits. Cooked foods were mainly vegetables consumed cold or stir-fried. Some plants were used as seasonings, infused wines, condiments and grains. The main medicinal functions were nourishing and reducing heatiness. Nourishing plants were mainly “shen” plants and Liliaceae, while plants able to reduce heatiness were mainly Asteraceae. Others functions included anti-hangover, anticancer and insecticidal. There were 38 species of important edible wild plants (CFSI > 500) in northern Guizhou, which had a high utilization rate. *Houttuynia cordata* Thunb. and *Mentha suaveolens* Ehrh. were the most representative edible wild plants in this area. The species, edible parts, edible categories, consumption modes and medicinal functions of edible wild plants in this area are diverse, and the traditional knowledge on their uses is rich. However, the number of wild plant species eaten by the informants and their related knowledge were positively correlated with age, which indicates that the rich traditional knowledge in this area is gradually disappearing with urbanization.

**Conclusions:**

The Gelao have a rich history of consuming wild plants. With the development of the social economy, the traditional knowledge passed from older generations is gradually being lost and its inheritance is facing great risks. This study collects, sorts and spreads this precious traditional knowledge, which is of great value to its protection and inheritance and fully demonstrates the value and importance of our work.

## Background

Wild plant resources play an indispensable role in the history of human development [1]. They are not only used to fill gaps in food supply caused by drought or resource shortages but also play an important role in maintaining the livelihood security of people in resource-deficient areas and in balancing the nutritional value of diets [2, 3]. With globalization, the food crisis has become prominent, and edible wild plant resources, especially those with a long tradition of use as food, will become an important supplementary food source for humans [4, 5].

The Gelao people are a unique minority in Southwest China, of whom more than 90% live in the northern part of Guizhou Province [6]. The mountainous geographical environment and abundant precipitation make this area rich in wildlife diversity [7], with many rare, endemic and ancient groups preserved. Northern Guizhou is one of the key land biodiversity areas in China given its high concentration of important biodiversity groups, which also has international significance [8, 9]. At the same time, the mountainous geography leads to a lack of sufficient cultivated land in this area. As a result, abundant wild plant resources have become an important supplementary food source for the Gelao people [10]. Over their long history, the Gelao people, combining their environmental conditions, religious beliefs and cultural customs, formed a unique traditional food culture and accumulated rich traditional knowledge on the utilization of wild plant resources [11]. This traditional knowledge on the available wild plant resources has a great influence on the protection and sustainable development and utilization of regional biodiversity [12, 13]. However, the Gelao people have no written language, and their traditional culture is thus mainly spread by word of mouth [6]. This mode of communication is easily thwarted by urbanization. With the rapid development of China's economy and information technology, the relocation of ethnic minorities is also accelerating, and the rich ethnic knowledge accumulated for thousands of years by ethnic minorities without their own written language is rapidly disappearing [14, 15]. This is no exception for the Gelao nationality. Therefore, a new way for communicating the traditional knowledge of the Gelao people is needed.

Through ethnobotany research, we can understand the local knowledge of Gelao people regarding the traditional use of plants and the relationship between Gelao people and their living environment in order to retain and pass on this traditional knowledge forever. At the same time, we can also explore wild plant resources with high utilization value, discuss their development value and provide appropriate suggestions for protecting biodiversity and sustainable development and utilization of wild resources in minority areas.

## Materials and methods

### Study area

**Fig. 1:**
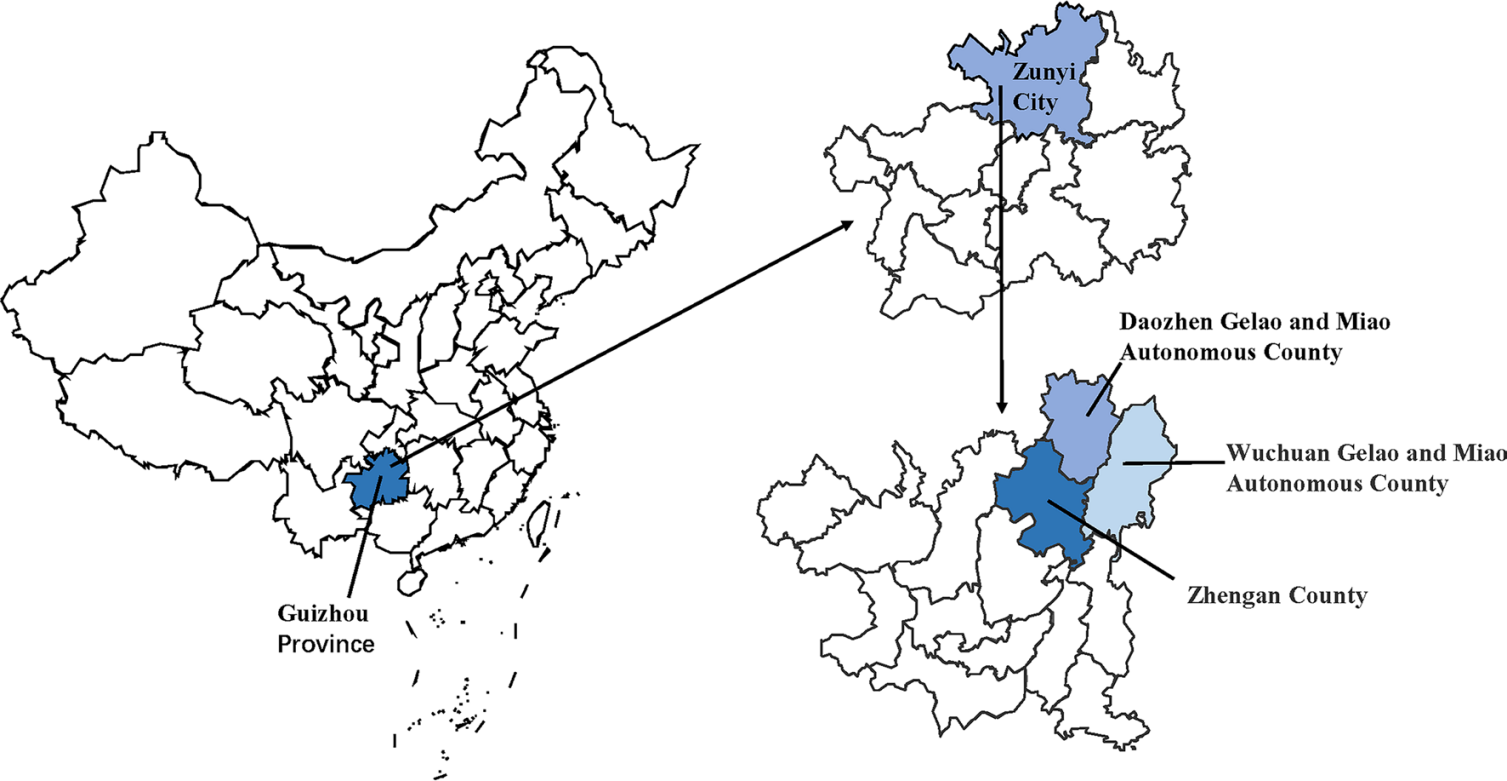
Survey area. Daozhen County, Wuchuan County and Zheng'an County belong to a minority autonomous county in the northeastern mountainous area of Guizhou Province

In this study, Daozhen County, Wuchuan County and Zheng 'an County in northern Guizhou are taken as the study areas (Fig. 1). This region spans 28°9′ to 29°13′ N, 107°4′ to 108°13′ E. It is located in the southeast, middle and east of Dalou Mountain and the upper reaches of the Furong River. It has a subtropical humid monsoon climate and a mid-subtropical humid monsoon climate; its average annual temperature is 8–16.14 °C, and the annual precipitation is 800–1400 mm. This area is a multiethnic settlement, and the main Chinese ethnic groups are the Gelao, Miao and Han (Table 1). Typical traditional agriculture in mountainous areas and industrial parks is the mainstay, and the main crops are corn, rice, potato, tea, pepper, *Chimonobambusa quadrangularis* (Franceschi) Makino and other Chinese herbal medicines, such as *Codonopsis radix*, *Bletilla striata* Rchb. f. and *Pseudocydonia* (C. K. Schneid.) C. K. Schneid. This area is located in the intersection zone between Guizhou and Chongqing, which is an important economic and cultural intersection area between southern Chongqing and northern Guizhou and has developed a unique diversified local culture.

**Table 1:** Basic information of study areas

County	Location	Population	Main ethnic	Main language	GDP/person	Investigation site	Longitude and latitude
Daozhen	North of Guizhou Province (E 107° 21′–107° 51′; N 28° 36′–29° 13′)	240,000	Glao (48%)/Han/Miao/Tujia	Chinese/Miao/Gelao	¥34,000	Sanlong village, Longxing town, Daoxian county	E 107° 22′; N 28° 42′
						Luolong village, Longxing town, Daoxian county	E 107° 41′; N 29° 3′
						Zhaoshan village, Zhongping town, Daoxian county	E 107° 41′; N 28° 41′
Wuchuan	North of Guizhou Province E 107° 30′–108° 13′; N 28° 11′–29° 05′	480,000	Glao (44%)/Han/Miao	Chinese/Miao/Gelao	¥360,00	Shankeng village, Daping town, Wuchuan county	E 108° 2′; N 28° 37′
						Tongxin village, Maotian town, Wuchuan county	E 108° 5′; N 28° 54′
						Shanshui village, Duluo town, Wuchuan county	E 107° 53′; N 28° 29′
Zhengan	North of Guizhou Province E 107° 4′–107° 41′; N 28° 9′–28° 51′	660,000	Han (65%)/Glao/Miao	Chinese/Miao/Gelao	¥350,00	Guangda village, Gelin town, Zhengan county	E 107° 30′; N 28° 37′
						Shiyin village, Miliang town, Zhengan county	E 107° 25′; N 28° 23′

### Ethnobotanical information collection

In the field investigation process, key-person interviews, semistructured interviews and participatory rural evaluation methods were adopted, and the basic content of interviews followed the “5W + 1H” question pattern [16]. This helped to uncover the traditional knowledge of edible wild plants and record, sort out and analyze the basic information provided by informants as well as the local common names, edible parts, edible categories, consumption modes and medicinal functions of edible plants.

The participatory observation method was used [17] to understand the species, uses, functions, edible parts and edible methods of wild plants collected and eaten in the daily life of the local people.

Video telephone interviews were also conducted, and the interview content was the same as that of the field survey.

### Ethnobotanical quantitative evaluation method

The cultural food significance index (CFSI) was used to evaluate the edible wild plants in this area.

$${\text{CFSI}} = {\text{FQI}} \times {\text{AI}} \times {\text{FUI}} \times {\text{PUI}} \times {\text{MFFI}} \times {\text{TSAI}} \times {\text{FMRI}} \times {1}0^{{ - {2}}}$$

where FQI is the frequency of quotation index, AI is the commonness index, FUI is the frequency of utilization index, PUI is the parts used index, MFFI is the multifunctional food use index, TSAI is the taste score appreciation index, and FMRI is the food medicinal role index [18].

According to the Common Research Methods of Ethnobotany [17], these indices are graded and assigned as follows: Frequency of quotation index (FQI): the number of people who mentioned a plant among all informants; Availability index (AI): divided into very common (4.0), common (3.0), average (2.0) and uncommon (1.0); Frequency of utilization index (FUI): divided into more than once a week (5.0), once a week (4.0), once a month (3.0), more than once a year but less than once a month (2.0), once a year (1.0) and unused for nearly 30 years (0.5); Parts used index (PUI): divided into whole plant (4.00), overground and underground parts (3.00), tender leaves and stems and leaves (2.00), flowers and fruits (1.50), tender roots, stems and stipules (1.00) and buds (0.75); Multifunctional food use index (MFFI): divided into raw food and cold salad (1.5), boiling, stewing and seasoning (1.0), special purpose and condiments (0.75) and raw food as snacks (0.50); Taste score evaluation index (TSAI): divided into excellent (10.0), very good (9.0), good (7.5), fair (6.5), poor (5.5) and very poor (4.5); Food-medicinal role index (FMRI): divided into very high (as medicinal food: 5.0), high (as medicine to treat a certain disease: 4.0), moderately high (very healthy food: 3.0), moderately low (healthy food, unknown efficacy: 2.0) and unknown or possibly toxic (1.0).

### Specimen identification

In the process of investigation, we collected the first recorded specimens and recorded the collection time, detailed place names (including latitude, longitude and altitude), and local and Latin names of the plants. Specimens were identified based on the electronic version of the full text of the Flora of China (http://www.iplant.cn/frps) [19], the Illustration of Flowering Plants in Hengduan Mountain [20] and the Field Identification Manual of Common Plants in China, Hengshan Book [21]. Plants collected during the study were identified to the species level, specimens were prepared and sorted, and collected information was analyzed and visualized using charts. Voucher Specimen numbers are provided in Table 2, and the specimens were deposited in the Life Science Museum and Pharmacognosy Teaching and Research Section of Zunyi Medical University.

**Table 2:** List of wild edible and healthy plants of Gelao minority residents in northern Guizhou

Families and genera	Scientific name	Local name in Chinese	Local name in pinyin	Plant type	Edible part	Food category	Edible method	Health care function	Voucher numbers
Amaryllidaceae	*Allium macranthum* Baker	苦蒜	KuSuan	Perennial herb	Whole grass/Bulb	Vegetables	Stir-fry vegetables with whole plant/Cold salad,Kimchi bulb	–	GZ-2022-ZA-211
	*Allium chrysanthum* Regel	野葱	YeCong	Perennial herb	Whole grass/Leaf	Vegetables	Stir-fry vegetables with whole plant/cold salad	–	GZ-2022-DZ-007
Asparagaceae	*Asparagus cochinchinensis* (Lour.) Merr	天冬	TianDong	Perennial herb	Root tuber	Health foods	Soak in wine	Nourishing	GZ-2022-ZA-031
	*Ophiopogon japonicus* (Thunb.) Ker Gawl	麦冬	MaiDong	Perennial herb	Root tuber	Health foods	Soak in wine	Nourishing	GZ-2022-ZA-109
	*Polygonatum sibiricum* Delar. ex Redoute	老虎姜	LaoHuJiang	Perennial herb	Root tuber	Health foods	Soak in wine	Nourishing	GZ-2022-ZA-225(P)
	*Polygonatum odoratum (Mill.) Druce*	玉竹	YuZhu	Perennial herb	Root tuber	Health foods	Soak in wine	Nourishing	GZ-2022-ZA-304(P)
Liliaceae	*Lilium brownii* F.E.Br. ex Miellez	百合	BaiHe	Perennial herb	Bulb	Vegetables/health foods	Soak in wine	Nourishing	GZ-2022-ZA-018
Asphodelaceae	*Hemerocallis citrina* Baroni	黄花菜	HuangHuaCai	Perennial herb	Bud	Vegetables	Fried vegetables/soup	–	GZ-2022-DZ-055
Smilacaceae	*Smilax china var. china*	土茯苓	TuFuLing	Perennial vine	Bud	Vegetables	Fried vegetables	–	GZ-2022-ZA-032
	*Smilax altissima* Roxb			Perennial vine	Bud	Vegetables	Fried vegetables	–	GZ-2022-ZA-038
Cupressaceae	*Platycladus orientalis* (L.) Franco	柏香	BaiXiang	Arbor	Shoot	Auxiliary food	Bacon	–	GZ-2022-ZA-114
Lamiaceae	*Perilla frutescens* (L.) Britton	紫苏	ZiSu	Perennial herb	Tender leaf	Seasoning vegetable	Soup/hot pot	expelling Summer-heat	GZ-2022-DZ-046
	*Perilla frutescens* (L.) Britton	白苏	BaiSu	Perennial herb	Tender leaf	Seasoning vegetable	Soup/hot pot	expelling Summer-heat	GZ-2022-DZ-052
	*Agastache rugosa* Kuntze	藿香	HuoXiang	Perennial herb	Tender leaf	Seasoning vegetable	Stewed crucia with agastache	Kaiwei	GZ-2022-DZ-003
	*Mentha suaveolens* Ehrh	鱼香/薄荷	YuXiang/BoHe	Perennial herb	Tender leaf	Seasoning vegetable	Boiled fish/boiled noodles	Kaiwei	GZ-2022-DZ-005
	*Mentha haplocalyx* Briq	薄荷	BoHe	Perennial herb	Tender leaf	Seasoning vegetable	Boiled fish (use less instead of Magnolia incense)	Kaiwei/expelling Summer-heat	GZ-2022-DZ-017
	*Stachys affinis* Bunge	地蚕/地葫芦	DiCan/DiHuLu	Perennial herb	Root tuber	Vegetables	Cold and dressed with sauce/fried vegetables	–	GZ-2022-ZA-034
Aquifoliaceae	*Ilex kudingcha* C.J.Tseng	苦丁茶	KuDingCha	Arbor	Leaf	Tea	Make tea (a special bitter taste called Kuding tea)	Clearing fire	GZ-2022-WC-053
Fabaceae	*Pueraria lobata* (Willd.) Ohwi	葛根	GeGen	Perennial woody vine	Root	Health foods	Soup/Pastries/Cooking porridge/Pueraria lobata (Willd.) Ohwi root powder (Extract Pueraria lobata (Willd.) Ohwi starch)	Hangover	GZ-2022-DZ-011
	*Vicia gigantea* Bunge	革命菜	GeMingCai	Annual herb	Bud/Tender leaf	Vegetables	Cold and dressed with sauce/fried vegetables	–	GZ-2022-WC-031
	*Robinia pseudoacacia* L	洋槐	YangHuai	Arbor	Flower	Vegetables/health foods	Fresh food	–	GZ-2022-ZY-002
Ericaceae	*Rhododendron simsii* Planch	杜鹃花	DuJuanHua	Shrub	Flower	Snack/vegetables	Fresh food/fried vegetables/soup	–	GZ-2022-DZ-053
	*Vaccinium bracteatum* Thunb	冷饭果	LengFanGuo	Small arbor	Fruit	Snack	Fresh food/soak in wine	–	GZ-2022-WC-049
Polyporaceae	*Ganoderma lucidum* (Leyss. Ex Fr.) Karst	灵芝	LingZhi	Fungus	Fruiting body	Health foods	Soup	Anticancer	GZ-2022-WC-097
Poaceae	*Lophatherum gracile* Brongn	地竹子	DiZhuZi	Perennial herb	Aboveground part	Tea	Make tea	Clearing fire	GZ-2022-WC-112
	*Phyllostachys edulis* J.Houz	楠竹	NanZhu	Arbor	Bud	Vegetables	Fried vegetables/pickle/drying	–	GZ-2022-WC-117
	*Phyllostachys bambusoides* f. *lacrima-deae* Keng f. & T.H.Wen	斑竹	BanZhu	Arbor	Bud	Vegetables	Fried vegetables/pickle/drying (stewed pork chops /pork hooves with bamboo shoots)	–	GZ-2022-WC-125
	*Dendrocalamus tsiangii* (McClure) Chia et H. L. Fung	钓鱼竹	DiaoYuZhu	Arbor	Bud	Vegetables	Fried vegetables/pickle/drying	–	GZ-2022-WC-144
	*Chimonobambusa quadrangularis* (Fenzi) Makino	方竹笋	FangZhuSun	Small arbor	Bud	Vegetables	Fresh stir-fried/drying	–	GZ-2022-ZA-132
	*Indocalamus tessellatus* (Munro) Keng f	粽子叶	ZongZiYe	Perennial herb	Leaf	Auxiliary food	For making zongzi	–	GZ-2022-WC-015
	*Phragmites australis* (Cav.) Steud	芦苇	LuWei	Perennial herb	Leaf	Auxiliary food	For making zongzi	–	GZ-2022-DZ-102
	*Coix lacryma-jobi* L	苡仁	YiRen	Perennial herb	Seeds	Multigrain	Cooking porridge	Removing dampness	GZ-2022-WC-022
Taxaceae	*Taxus wallichiana* var. *chinensis* (Pilg.) Florin	红豆杉	HongDouShan	Arbor	Fruit	Medicinal materials	Soak in wine	Anticancer	GZ-2022-ZA-030
Juglandaceae	*Carya cathayensis* Sarg	核桃/胡桃	HeTao/HuTao	Arbor	Fruit/Bud	Vegetables/snack/nut	Stir-fried vegetables with sprouts/fresh fruit, dry food, fresh nut salad	Nourishing	GZ-2022-WC-001
Elaeagnaceae	*Elaeagnus pungens* Thunb	羊奶子/羊咪咪	YangNaiZi/YangMiMi	Shrub	Fruit	Snack	Fresh food	–	GZ-2022-ZA-017
Cucurbitaceae	*Gynostemma pentaphyllum* (Thunb.) Makino	绞股蓝	JiaoGuLan	Perennial twining herb	Leaf	Tea	Make tea	Nourishing	GZ-2022-WC-056
Zingiberaceae	*Zingiber striolatum* Diels	阳藿/阳荷	YangHuo/YangHe	Perennial herb	Bud	Vegetables	Cold and dressed with sauce/fried vegetables	–	GZ-2022-WC-093
Violaceae	*Viola betonicifolia* J. E. Smith	紫花地丁	ZiHuaDiDing	Perennial herb	Bud	Vegetables	Cold and dressed with sauce/fried vegetables	–	GZ-2022-WC-007
Malvaceae	*Malva cathayensis* M.G.Gilbert, Y.Tang & Dorr	葵菜	KuiCai	Perennial herb	Tender leaf	Vegetables	Fried vegetables	–	GZ-2022-WC-106
Crassulaceae	*Sedum emarginatum* Migo	豁叶菜	HuoYeCai	Perennial herb	Bud	Vegetables	Cold and dressed with sauce/fried vegetables	–	GZ-2022-WC-123
	*Sedum sarmentosum* Bunge	石头菜	ShiTouCai	Perennial herb	Bud	Vegetables	Cold and dressed with sauce/fried vegetables	–	GZ-2022-WC-127
	*Sedum fui* G.D.Rowley	肺心菜	FeiXinCai	Perennial succulent herb	Stem/Leaf	Vegetables	Cold and dressed with sauce/fried vegetables/make noodles	Nourishing (Nourishing cardiopulmonary function)	GZ-2022-ZA-016
Campanulaceae	*Adenophora stricta* Miq	泡参	PaoShen	Perennial herb	Root	Health foods	Daube/soak in wine	Nourishing	GZ-2022-DZ-033
	*Diapensia bulleyana* Forrest ex Diels	党参/臭参	DangShen/ChouShen	Perennial herb	Root	Health foods	Daube/soak in wine	Nourishing	GZ-2022-DZ-034
	*Codonopsis tubulosa* Kom	党参/臭参	DangShen/ChouShen	Perennial herb	Root	Health foods	Daube/soak in wine	Nourishing	GZ-2022-DZ-036
	*Codonopsis pilosula subsp. tangshen* (Oliv.) D.Y.Hong	党参/臭参	DangShen/ChouShen	Perennial herb	Root	Health foods	Daube/soak in wine	Nourishing	GZ-2022-DZ-039
	*Codonopsis pilosula* Nannf	党参/臭参	DangShen/ChouShen	Perennial herb	Root	Health foods	Daube/soak in wine	Nourishing	GZ-2022-DZ-041
	*Campanumoea javanica* Blume	土党参	TuDangShen	Perennial herb	Root	Health foods	Daube/soak in wine	Nourishing	GZ-2022-WC-104
Asteraceae	*Aster indicus* L	马兰头	MaLanTou	Perennial herb	Bud	Vegetables	Fried vegetables/cold and dressed with sauce	Clearing fire	GZ-2022-DZ-015
	*Sonchus brachyotus* DC	苦菜	KuCai	Perennial herb	Bud	Vegetables	Fried vegetables/cold and dressed with sauce	–	GZ-2022-WC-002
	*Crassocephalum crepidioides* (Benth.) S.Moore	野茼蒿	YeTongHao	Perennial herb	Bud	Vegetables	Fried vegetables/cold and dressed with sauce	–	GZ-2022-DZ-013
	*Gnaphalium affine* D.Don	清明菜	QingMingCai	Annual herb	Bud	Vegetables	Ba (Fig. 3)	–	GZ-2022-DZ-016
	*Artemisia indica* Willd	野艾	YeAi	Perennial herb	Bud/Tender leaf	Vegetables	Aimomo (Fig. 3)	–	GZ-2022-DZ-019
	*Artemisia lavandulifolia* DC	野艾	YeAi	Perennial herb	Bud/Tender leaf	Vegetables		Clearing fire	GZ-2022-DZ-024
	*Artemisia argyi* H.Lév. & Vaniot	艾蒿/艾草	AiHao/AiCao	Perennial herb	Bud/Tender leaf	Vegetables		–	GZ-2022-DZ-026
	*Taraxacum sect. Erythrocarpa* Hand.-Mazz	蒲公英	PuGongYing	Perennial herb	Bud/Tender leaf	Vegetables	Cold and dressed with sauce/hot pot	Clearing fire	GZ-2022-DZ-008
	*Dendranthema indicum* (L.) Des Moul	野菊花	YeJuHua	Perennial herb	Inflorescence	Health foods	Soak wine/make tea	Clearing fire	GZ-2022-WC-014
	*Cirsium arvense var. integrifolium* Wimm. & Grab	刺儿菜	CiErCai	Perennial herb	Bud	Vegetables	Fried vegetables/cold and dressed with sauce	–	GZ-2022-WC-096
	*Aster trinervius subsp. ageratoides* (Turcz.) Grierson	柴胡	ChaiHu	Perennial herb	Bud	Vegetables	Fried vegetables/cold and dressed with sauce	Mistakenly made Bupleurum chinense	GZ-2022-DZ-044
	*Youngia japonica* (L.) DC	小苦菜	XiaoKuCai	Perennial small herb	Bud/Tender leaf	Vegetables	Cold and dressed with sauce/fried vegetables	–	GZ-2022-DZ-145
	*Sonchus wightianus* DC	大苦菜	DaKuCai	Perennial herb	Bud/Tender leaf	Vegetables	Cold and dressed with sauce/fried vegetables	–	GZ-2022-DZ-127
	*Ixeris polycephala* Cass	大苦菜	DaKuCai	Perennial small herb	Bud/Tender leaf	Vegetables	Cold and dressed with sauce/fried vegetables	–	GZ-2022-DZ-045
	*Ixeris chinensis* (Thunb.) Nakai	小苦菜	XiaoKuCai	Perennial herb	Bud/Tender leaf	Vegetables	Cold and dressed with sauce/fried vegetables	–	GZ-2022-DZ-020
	*Symphyotrichum subulatum* (Michx.) G.L.Nesom	土柴胡	TuChaiHu	Perennial herb	Bud	Vegetables	Cold and dressed with sauce/fried vegetables	–	GZ-2022-DZ-032
Fagaceae	*Castanea mollissima* Blume	毛栗	MaoLi	Arbor	Seeds	Snack	Raw food/fried food	Nourishing (Nourishing asphyxia)	GZ-2022-WC-012
	*Lithocarpus litseifolius* (Hance) Chun	甜茶	TianCha	Arbor	Leaf	Tea	Make tea (special sweetness, called sweet tea)	expelling Summer-heat	GZ-2022-ZA-026
Orchidaceae	*Gastrodia elata* Blume	天麻/赤箭	TianMa/ChiJian	Perennial herb	Tuber	Supplements	Soup/daube/soak in wine	Treatment migraine	GZ-2022-ZA-046
Chenopodiaceae	*Chenopodium album* L	灰灰菜	HuiHuiCai	Annual herb	Bud	Vegetables	Fried vegetables/cold and dressed with sauce	–	GZ-2022-DZ-001
Meliaceae	*Toona sinensis* (A. Juss.) Roem	香椿	XiangChun	Arbor	Bud	Vegetables	Fried vegetables/cold and dressed with sauce	–	GZ-2022-WC-017
Polygonaceae	*Reynoutria japonica* Houtt	酸汤梗	SuanShangGeng	Perennial herb	Bud	Vegetables	Fried vegetables/cold and dressed with sauce	–	GZ-2022-ZA-010
	*Pleuropterus multiflorus* Turcz. ex Nakai	何首乌	HeShouWu	Perennial herb	Root tuber	Snack/Health foods	Eat directly after cooking/soup	Nourishing	GZ-2022-ZA-003
	*Fagopyrum dibotrys* (D.Don) Hara	野兰荞	YeLanQiao	Perennial herb	Bud	Vegetables	Fried vegetables/cold and dressed with sauce	–	GZ-2022-DZ-038
Basellaceae	*Anredera cordifolia* (Ten.) Steenis	豆腐菜	DouFuCai	perennial grassy vine	Tender leaf	Vegetables	Fried vegetables/boiled noodles	–	GZ-2022-WC-072
	*Basella alba* L	软浆子	RuanJiangZi	perennial grassy vine	Tender leaf	Vegetables	Fried vegetables/boiled noodles	–	GZ-2022-WC-083
Verbenaceae	*Premna microphylla* Turcz	豆腐柴	DouFuChai	Shrub	Leaf	Vegetables	Tofu (Fig. 5)	–	GZ-2022-DZ-078
	*Vitex negundo* L	黄荆条	HuangJingTiao	Shrub	Stem/Leaf	Auxiliary food	Sauce	–	GZ-2022-ZA-096
Portulacaceae	*Portulaca oleracea* L	马齿苋	MaChiXian	Annual herb	Whole grass	Vegetables	Cold and dressed with sauce/fried vegetables	Treatment diarrhea	GZ-2022-DZ-088
	*Talinum paniculatum* (Jacq.) Gaertn	土人参	TuRenShen	Perennial herb	Tender leaf / Root	Vegetables/Medicinal materials	Stir-fried vegetables with sprouts/root soup	Nourishing	GZ-2022-DZ-014
Actinidiaceae	*Actinidia chinensis* Planch	马屎蛋/羊桃	MaShiDan/YangTao	Woody vine	Fruit	Fruits	Fresh food	–	GZ-2022-DZ-154(P)
Auriculariacaee	*Auricularia polytricha* (Mont.) Sacc	木耳	MuEr	Fungus	Fruiting body	Vegetables	Cold and dressed with sauce/fried vegetables	–	GZ-2022-WC-021(P)
Schisandraceae	*Schisandra chinensis* (Turcz.) Baill	秤砣子	ChengTuoZi	Woody vine	Fruit	Snack	Fresh food/soak in wine	–	GZ-2022-DZ-079
Lardizabalaceae	*Akebia trifoliata* (Thunb.) Koidz	八月瓜/八月炸	BaYueGua/BaYueZha	Perennial vine	Fruit	Snack	Fresh food	–	GZ-2022-DZ-083
Oleaceae	*Osmanthus fragrans* Lour	桂花	GuiHua	Arbor	Flower	Wines	Wine	–	GZ-2022-ZA-049
	*Ligustrum quihoui* Carrière	苦丁茶	GuDingCha	Small arbor	Leaf	Tea		–	GZ-2022-DZ-077
Nostocaceae	*Nostoc commune* Vaucher	地耳子	DiErZi	Fungus	Fruiting body	Vegetables	Scrambled egg/steamed stuffed bun	–	GZ-2022-WC-035
Boletaceae	*Boletus edulis* Fr	大脚菇	DaJiaoGu	Fungus	Fruiting body	Vegetables	Fried vegetables/soup	–	GZ-2022-DZ-031(P)
	*Morchella esculenta* (L.) Pers	黄癞头	HuangLaiTou	Fungus	Fruiting body	Vegetables	Fried vegetables/soup	–	GZ-2022-DZ-035(P)
Araliaceae	*Aralia elata *(Miq.) Seem	狼牙棒	LangYaBang	Small arbor	Bud	Vegetables	Fried vegetables	–	GZ-2022-WC-016
Rosaceae	*Rosa roxburghii* Tratt	刺梨	CiLi	Large shrub	Fruit	Snack/Wines	Fresh food/dried fruit/soak in wine/non-alcoholic beverages	Hangover	GZ-2022-DZ-073
	*Chaenomeles japonica* (Thunb.) Lindl. ex	野木瓜	YeMuGua	Small arbor	Fruit	Snack	Fresh food/dried fruit/preserved fruit	Hangover	GZ-2022-ZA-028(P)
	*Chaenomeles speciosa* (Sweet) Nakai	野木瓜	YeMuGua	Small arbor	Fruit	Snack	Dried fruit/preserved fruit	Hangover	GZ-2022-ZA-029(P)
	*Rosa laevigata* Michx	TangLang果/金樱子	TangLangGuo/JinYingZi	Perennial vine shrub	Fruit	Snack/Medicinal materials	Soak in wine	Nourishing (Nourishing yangqi)	GZ-2022-WC-109
	*Pyracantha fortuneana* (Maxim.) H.L.Li	红籽/救命粮/救兵粮	HongZi/JiuMingLiang/JiuBingLiang	Small arbor	Fruit/Leaf	Snack/Medicinal materials	Fresh food/soak in wine	–	GZ-2022-ZA-013
	*Duchesnea filipendula* Focke	蛇泡儿	ShePaoEr	Perennial herb	Fruit	Snack	Fresh food	–	GZ-2022-ZA-040
	*Fragaria vesca* L	白米泡	BaiMiPao	Perennial herb	Fruit	Snack	Fresh food	–	GZ-2022-ZA-037
	*Rubus coreanus* Miq	栽秧泡	ZaiYangPao	Perennial semi-vine shrub	Fruit	Snack	Fresh food/soak in wine	–	GZ-2022-ZA-041
	*Rubus inopertus* (Focke ex Diels) Focke	黄泡	HuangPao	Perennial semi-vine shrub	Fruit	Snack	Fresh food/soak in wine	–	GZ-2022-ZA-091
	*Rubus pluribracteatus* L.T.Lu & Boufford	乌泡	WuPao	Perennial semi-vine shrub	Fruit	Snack	Fresh food/soak in wine	–	GZ-2022-ZA-098
	*Rubus parvifolius* L	酸泡	SuanPao	Perennial semi-vine shrub	Fruit	Snack	Fresh food/soak in wine (sour)	–	GZ-2022-ZA-103
	*Rubus idaeus* L	覆盆子	FuPenZi	Perennial semi-vine shrub	Fruit	Snack	Fresh food/soak in wine (sour)	Nourishing (Nourishing yangqi)	GZ-2022-ZA-107
	*Carex xerophila* Janeway & Zika	野梨	YeLi	Arbor	Fruit	Snack	Fresh food	–	GZ-2022-WC-113
Solanaceae	*Nicandra physalodes* (L.) Gaertn	冰粉籽	BingFenZi	Annual herb	Seeds	Feature foods	Ice powder	–	GZ-2022-DZ-143
	*Solanum nigrum* L	黑星星	HeiXingXing	Annual herb	Bud/Fruit	Vegetables/Snack	Fried vegetables or fresh food	–	GZ-2022-DZ-142
	*Atropa bella-donna* L	刺茄子	CiQieZi	Perennial herb	Fruit	Snack	Fresh food (after fresh, frost, green fruit is poisonous)	–	GZ-2022-ZA-007
	*Alkekengi officinarum* Moench	灯笼果	DengLongGuo	Shrub	Fruit	Snack	Fresh food/soak in wine	Clearing heat and removing swelling	GZ-2022-WC-154
Caprifoliaceae	*Lonicera macrantha* Spreng	金银花	JinYinHua	Perennial woody vine	Flower	Tea/Medicinal materials	Make tea	Clearing fire	GZ-2022-ZA-033
	*Lonicera humilis* Kar. & Kir			Perennial woody vine	Flower	Tea/Medicinal materials	Make tea	Clearing fire	GZ-2022-DZ-132
	*Lonicera hypoglauca* Miq			Perennial woody vine	Flower	Tea/Medicinal materials	Make tea	Clearing fire	GZ-2022-ZA-155
Saururaceae	*Houttuynia cordata* Thunb	折耳根	SheErGen	Perennial herb	Bud/Tender root	Vegetables	Cold and dressed with sauce/hot pot dip/fried vegetables (fried bacon)	Clearing fire (Prevent COVID-19)	GZ-2022-ZA-001
Apiaceae	*Hydrocotyle sibthorpioides* Lam	星叶菜	XingYeCai	Perennial voldemort small herb	Tender leaf	Vegetables	Scrambled egg	–	GZ-2022-DZ-133
	*Ligusticum sinense* var*. hupehense* H.D.Zhang	川芎	ChuanXiong	Perennial herb	Bud	Vegetables	Cold and dressed with sauce/fried vegetables	–	GZ-2022-DZ-198
	*Oenanthe javanica* DC	水芹菜	ShuiQinCai	Annual herb	Bud	Vegetables	Cold and dressed with sauce/fried vegetables	–	GZ-2022-ZA-157
	*Cryptotaenia japonica* Hassk	鸭脚板	YaJiaoBan	Annual herb	Bud	Vegetables	Fried vegetables/cold and dressed with sauce	–	GZ-2022-ZA-159
	*Cestrum inclusum* Urb	积雪草	JiXueCao	Perennial herb	Bud	Vegetables	Fried vegetables/cold and dressed with sauce	Cosmetic effect	GZ-2022-WC-003
Moraceae	*Broussonetia papyrifera* (L.) L'Hér. ex Vent			Arbor	Fruit	Snack	Fresh food	Nourishing	GZ-2022-DZ-177
	*Morus alba* L	马山泡	MaShanPao	Arbor	Fruit/Leaf	Snack/Tea	Fresh food/soak in wine/leaf tea	Fruit blood tonification/Leaf clearing fire	GZ-2022-WC-046
	*Ficus carica* L	无花果	WuHuaGuo	Arbor	Fruit	Snack	Fresh food/dried fruit	–	GZ-2022-WC-004
	*Ficus tikoua* Bureau	地枇杷	DiPiPa	Prostrate small shrub	Fruit	Snack	Fresh food	–	GZ-2022-DZ-012
Vitaceae	*Vitis amurensis* Rupr	野葡萄	YePuTao	Perennial woody vine	Fruit	Snack	Fresh food	–	GZ-2022-DZ-101
Phytolaccaceae	*Phytolacca americana* L	红参	HongShen	Perennial herb	Root	Health foods	Soup	Mistakenly made Panax ginseng C. A. Meyer	GZ-2022-ZA-004
Brassicaceae	*Capsella bursa-pastoris* (L.) Medik	地米菜	DiMiCai	Annual herb	Bud	Vegetables	Cold and dressed with sauce	–	GZ-2022-DZ-002
Ebenaceae	*Diospyros lotus* Lour	野柿子	YeShiZi	Small arbor	Fruit	Snack	With sauce	–	GZ-2022-DZ-192
	*Diospyros kaki* L.f	野柿子	YeShiZi	Arbor	Fruit	Snack	Fresh food/soak in wine	–	GZ-2022-DZ-157
Rhamnaceae	*Hovenia acerba* Lindl	拐枣	GuaiZao	Arbor	Fruit	Fruits	Fresh food	–	GZ-2022-WC-111
Dioscoreaceae	*Dioscorea japonica* Thunb	山药	ShanYao	Perennial twining herb	Root	Vegetables	Cold salad or direct steaming after cooking	Tonifying Qi	GZ-2022-WC-009
	*Dioscorea nummularia* Lam	山药	ShanYao	Perennial twining herb	Root	Vegetables	Cold salad or direct steaming after cooking	Tonifying Qi	GZ-2022-DZ-122
	*Dioscorea nipponica* Makino	山药	ShanYao	Perennial twining herb	Root	Vegetables	Cold salad or direct steaming after cooking	Tonifying Qi	GZ-2022-WC-010
Pinaceae	*Pinus massoniana* Lamb	松树	SongShu	Arbor	Seeds	Snack	Fried food	–	GZ-2022-ZA-005
	*Pinus tabuliformis* Carrière	松树	SongShu	Arbor	Seeds	Snack	Fried food	–	GZ-2022-WC-005
Athyriaceae	*Callipteris esculenta* (Retz.) J.Sm	蕨菜	JueCai	Fern	Bud	Vegetables	Fresh stir-fried/Make dried vegetables after drying	–	GZ-2022-WC-013
Araceae	*Pinellia ternata* (Thunb.) Makino	麻芋子/三步跳	MaYuZi/SanBuTiao	Perennial herb	Tuber	Snack	Eat after steaming	Insecticidal	GZ-2022-ZA-118
	*Amorphophallus variabilis* Blume	野魔芋	YeMoYu	Perennial herb	Tuber	Snack	Eat after steaming	Insecticidal	GZ-2022-ZA-125
	*Amorphophallus konjac* K.Koch	魔芋	MoYu	Perennial herb	Tuber	Vegetables	MoYu (Fig. 5)	–	GZ-2022-WC-095
	*Colocasia antiquorum* Schott	广菜	GuangCai	Perennial herb	rhizome	Vegetables	Stew	–	GZ-2022-WC-098
Cactaceae	*Opuntia dillenii* (Ker Gawl.) Haw	仙人掌	XianRenZhang	Succulent shrub	Fruit/Fleshy leaf	Snacks on fruit/Fleshy leaves for vegetables	Fresh food/soak in wine/cold and dressed with sauce	–	GZ-2022-ZA-035
Amaranthaceae	*Amaranthus tricolor* L	红苋菜	HongHanCai	Perennial herb	Bud	Vegetables	Cold and dressed with sauce/fried vegetables	–	GZ-2022-ZA-039
Berberidaceae	*Epimedium borealiguizhouense* S.Z.He & Y.K.Yang	淫羊藿	YinYangHuo	Perennial herb	Whole grass/Leaf	Health foods	Make soup/soak in wine	Nourishing (Aphrodisiac effect)	GZ-2022-WC-066
Urticaceae	*Laportea bulbifera* (Siebold & Zucc.) Wedd	红活麻	HongHuoMa	Perennial herb	Bud	Vegetables	Fried vegetables	Treatment skin disease (Itching/Tinea)	GZ-2022-WC-073
	*Urtica fissa* E.Pritz. ex Diels	活麻	HuoMa	Perennial herb	Bud/Tender leaf	Vegetables	Hot pot		GZ-2022-WC-077
	*Gonostegia hirta* Miq	糯米条	NuoMiTiao	Shrub	Fruit	Snack	Fresh food/soak in wine	–	GZ-2022-WC-026
Myricaceae	*Morella rubra* Lour	野杨梅	YeYangMei	Arbor	Fruit	Fruits/Wines	Fresh food/brewing wine, soak in wine	–	GZ-2022-ZA-083
Melastomataceae	*Melastoma dodecandrum* Lour	地瓜	DìGua	Prostrate small shrub	Fruit	Snack	Fresh food	–	GZ-2022-WC-059
Tremellaceae	*Tremella fuciformis* Berk	银耳	YínEr	Fungus	Fruiting body	Auxiliary food	Boiled porridge (Tremella porridge)	Anticancer	GZ-2022-ZA-021(P)
Ginkgoaceae	*Ginkgo biloba* L	白果	BaiGuo	Arbor	Seeds	Vegetables/Medicinal materials	Daube/soak in wine	Nourishing (Nourishing asphyxia)	GZ-2022-DZ-004
Rutaceae	*Tetradium ruticarpum* (A. Jussieu) T. G. Hartley	臭花椒	ChouHuaJiao	Small arbor	Fruit	Condiment	Role of *Capsicum annuum* L	Activating blood circulation	GZ-2022-WC-091
	*Zanthoxylum simulans* Hance	野花椒	YeHuaJiao	Shrub	Fruit	Condiment	Role of *Zanthoxylum bungeanum* Maxim	–	GZ-2022-ZA-116
Lauraceae	*Litsea pungens* Hemsl	木姜子	MuJiangZi	Arbor	Seeds	Condiment	Stir-fry and press the oil as a seasoning	–	GZ-2022-WC-006
Osmundaceae	*Osmunda lancea* Thunb	蕨菜	JueCai	Fern	Bud	Vegetables	Fresh stir-fried/Make dried vegetables after drying	–	GZ-2022-ZA-036
Oxalidaceae	*Oxalis corniculata* L	醋浆子/酸浆子	CuJiangZi/SuanJiangZi	Perennial herb	Root tuber	Snack	Fresh food (sour)	–	GZ-2022-ZA-048
Russulaceae	*Russula vinosa* Lindblad	青头菌	QingTouJun	Fungus	Fruiting body	Vegetables	Fried vegetables/soup	–	GZ-2022-WC-023(P)
Tricholomataceae	*Collybia albuminosa* (Berk.) Petch	三把菇	SanBaGu	Fungus	Fruiting body	Vegetables	Fried vegetables/soup	–	GZ-2022-WC-024(P)

## Results

### Basic information from reports

**Fig. 2:**
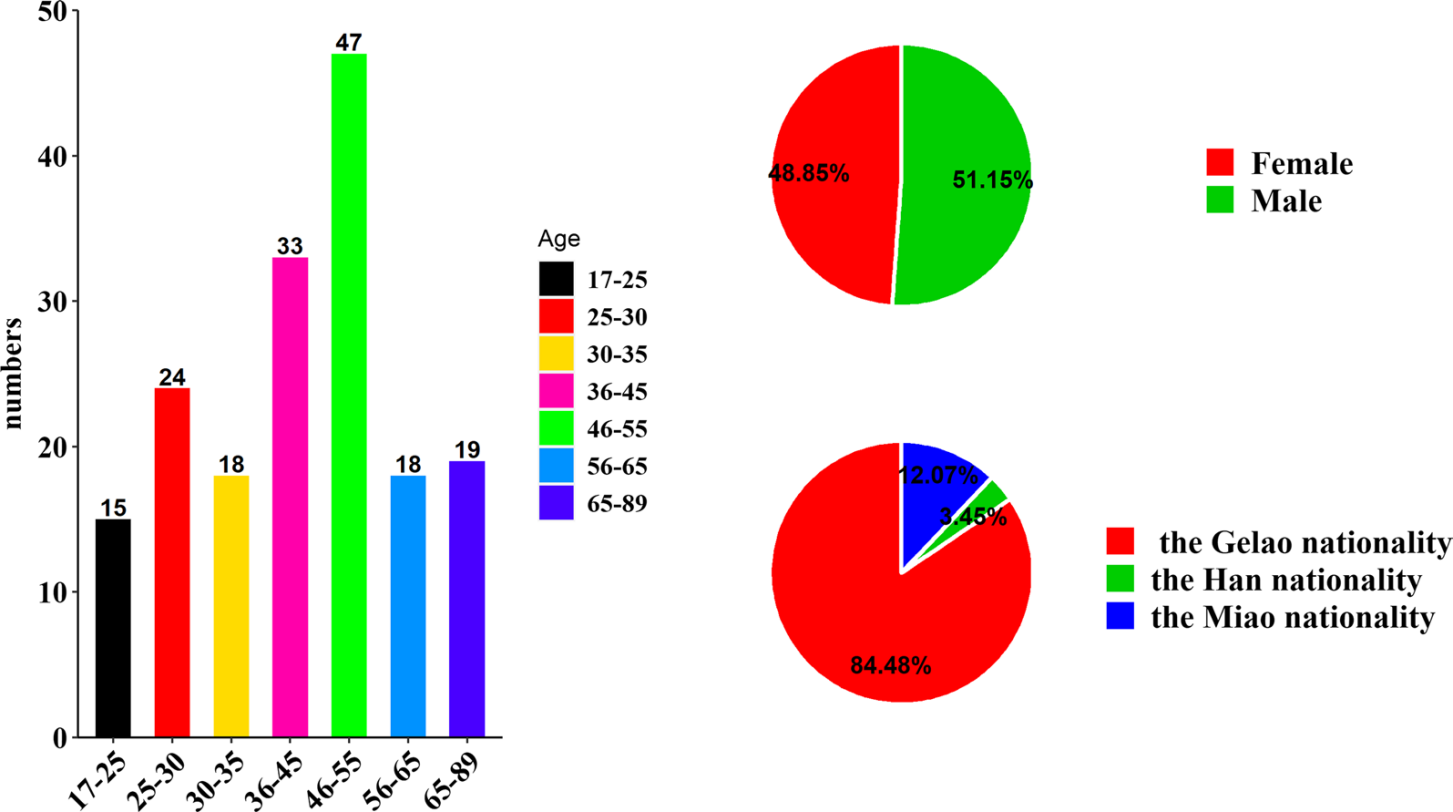
Basic information about the interviewees

The age distribution of the 174 informants was divided into age groups. The results showed that all informants were aged between 17 and 89, including 15 informants aged between 17 and 25, 24 between 26 and 30, 18 between 31 and 35, 33 between 36 and 45, 47 between 46 and 55, 18 between 56 and 6, and 19 between 65 and 89. There were 89 males and 85 females, with a male-to-female ratio of nearly 1:1. There were 147 informants of the Gelao nationality (accounting for 84.48% of the total), 21 of the Miao nationality and 6 of the Han nationality. The results show a positive correlation between the species of wild plants eaten by the reporter and age, and with the increase in the reporter's age, the number of edible plant species and corresponding information that can be provided were more abundant. This pattern is consistent with our earlier research on edible wild plant resources in Hasi Mountain [22]. A total of 16.40 species of wild plants have been eaten by 15 informants under the age of 25, most of which are common wild vegetables or fruits, and most of the informants only know the local names of plants and can provide less information about specific plants and eating methods. However, 19 informants over 65 years old have eaten as many as 66.05 kinds of wild plants per capita, which is 4.03 times that of informants under 25 years old, and some special knowledge is only known by elderly individuals, but no one has made use of this knowledge to prepare special foods, such as using wild fruit to make wine and using *Vitex negundo* L. and other plants as condiments to make sauce (Fig. 2).

### Sources of Gelao edible wild plants in northern Guizhou

**Fig. 3:**
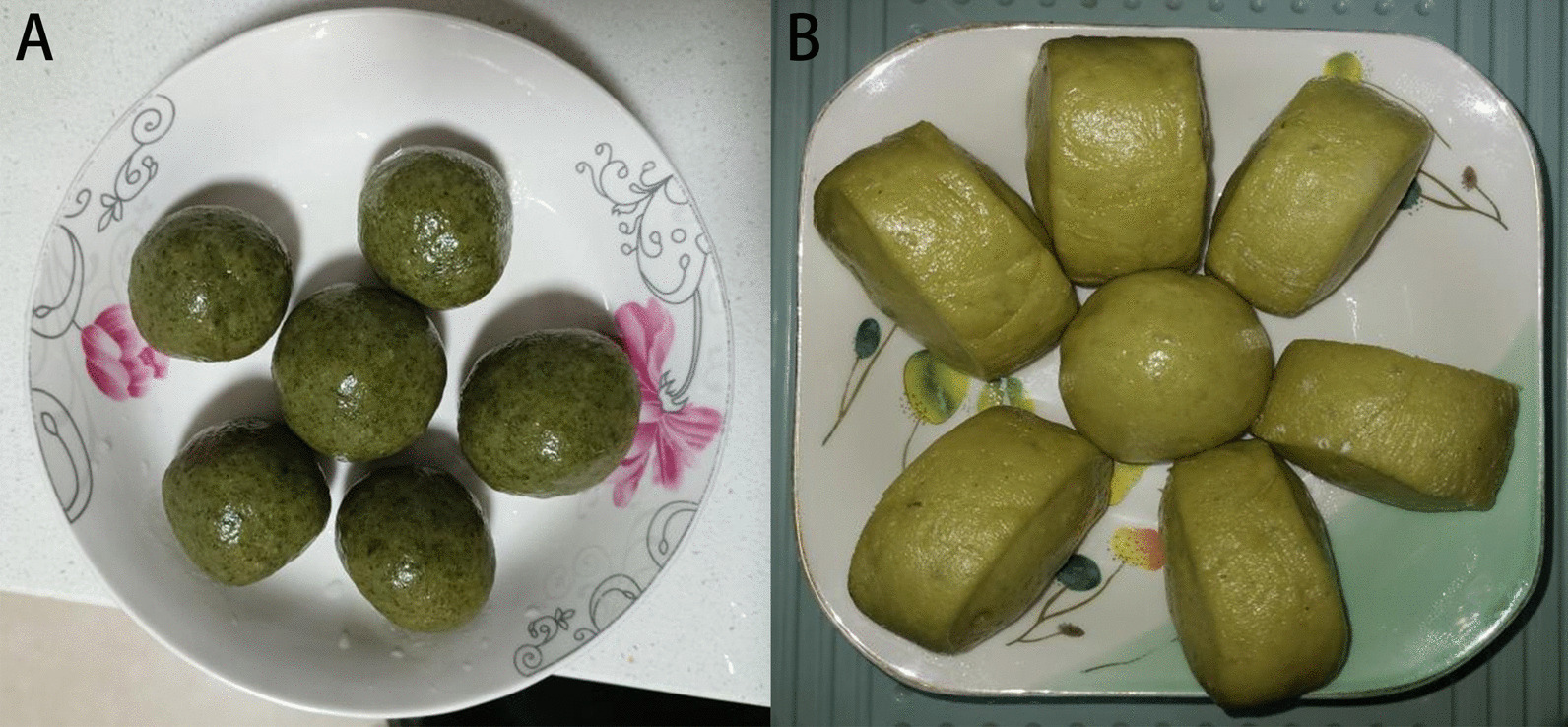
Buns made with *G. affine* D. Don (**A**) and *A. lavandulifolia* DC. (**B**). **A** is “Qingming”Ba which is made by flour and *G. affine* D. Don with the function of enhancing digestion. **B** is “Aimomo” which is made by flour and *A. lavandulifolia* DC. with the function of sterilization and digestion

**Fig. 4:**
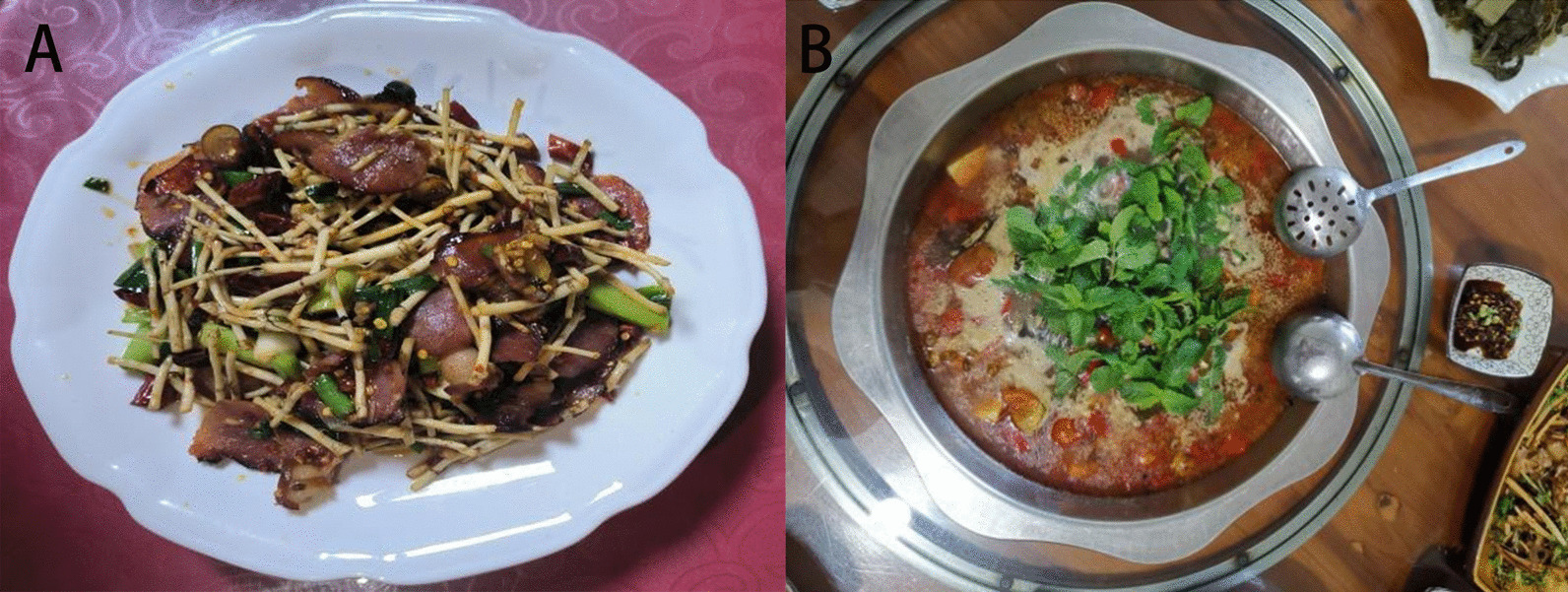
Stir-fried preserved pork with *H. cordata* Thunb. (**A**) and pork cooked with *M. suaveolens* Ehrh. (**B**). *H. cordata* Thunb. and *M. suaveolens* Ehrh. are usually used as seasonings of Gelao nationality, which have relieve inflammation and antiviral effects

The edible plants in Gelao people's residential areas in northern Guizhou were statistically analyzed. Incomplete statistics showed that there were 151 species (varieties) of traditional edible wild plants in this area, belonging to 67 families, with Asteraceae, Rosaceae and Poaceae being the most abundant families, with 16, 13 and 8 species, respectively (Table 2). Among the 16 species of edible wild plants in Asteraceae, the edible part of *Dendranthema indicum* (L.) Des Moul. is the inflorescence, whereas in all the other plants, the tender seedlings and leaves are the edible parts. Among these plants, several are mainly used to make a kind of food called “Ba,” such as *Gnaphalium affine* D. Don, *Artemisia lavandulifolia* DC., and *Artemisia indica* Willd (Fig. 3). Regarding the 13 edible wild plants in Rosaceae, the fruits of most are the edible part, and they are mainly consumed as snacks. Lamiaceae, Campanulaceae and Apiaceae also had a good number of edible species. Asparagaceae, Moraceae and Araceae each had four edible wild species. However, Araceae may contain more edible species, but it was difficult to distinguish them during the investigation (Fig. 4).

### Edible parts of Gelao edible wild plants in northern Guizhou

**Fig. 5:**
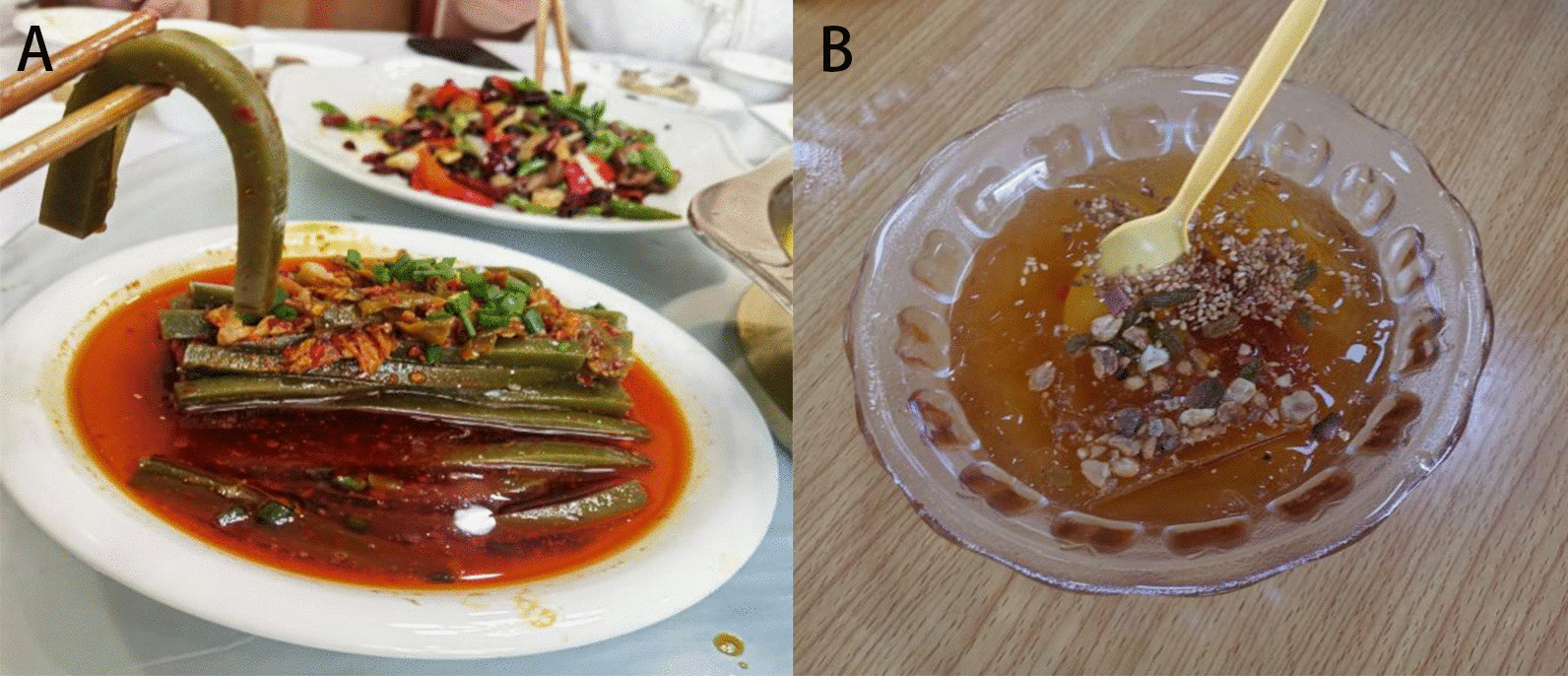
A kind of tofu made with *P. microphylla* Turcz. (**A**) and a beverage made with *N. physalodes* (L.) Gaertn. (**B**).** A** is made by adding plant ash into the juice kneaded from the leaves of *P. microphylla* Turcz.** B** is made from *N. physalodes* (L.) Gaertn. seeds by boiling and freezing

Among the 151 species of edible wild plants, fruits (including young fruits) were the most common edible parts, with 57 species. Trees and shrubs were the main types of plants, and they were most frequently consumed as snacks. Most of the preserved fruits have poor taste or are abundant but not easy to preserve, such as *Rosa roxburghii* Tratt., *Chaenomeles speciosa* (Sweet) Nakai, and *Ficus carica* L. The fruits used for making infused wine cannot be eaten directly, but they have specific medicinal functions, such as *Rosa laevigata* Michx. and *Taxus wallichiana var. chinensis* (Pilg.) Florin. There were 54 species of young stems and leaves (tender leaves, tender seedlings, tender buds) consumed, most of which were consumed cold or stir-fried. Most of the plant types are annual herbs or perennial herbaceous plants that die in autumn and winter and grow new buds in spring, represented by Asteraceae and Apiaceae. In addition, the edible parts included roots or rhizomes (bulbs), flowers, twigs, whole plants, and fruiting bodies. There were two consumption modes: raw and cooked. Raw foods were mainly consumed as snacks, which mainly included fruits. Cooked foods were mainly vegetables, which were mainly consumed cold or stir-fried with young stems and leaves. In addition, the edible wild plants were also used as seasoning, infused wine, condiments, miscellaneous grains, etc. Some plants had many edible parts, such as *Allium macranthum* Baker and *Pyracantha fortuneana* (Maxim.) H.L. Li. (Table 2). Some plants are named after the foods that they can be made into locally. For example, Doufuchai (*Premna microphylla* Turcz.) means a plant that can be made into tofu (Fig. 5), and Bing Fenzi (*Nicandra physalodes* (L.) Gaertn.) means that the seeds of this plant are mainly used to make a summer-heat-relieving drink.

### Medicinal function of Gelao edible wild plants in northern Guizhou

Among the 151 species of edible wild plants counted, there were 61 species that local residents believe have medicinal value in addition to edible value, accounting for 40.4% of the total (Table 2). Medicinal functions mainly included nourishing and reducing ‘heatiness,’ and for most of the nourishing plants, the roots were the edible parts. Local residents refer to the plant roots with nourishing effects as "shen," such as tangshen (*Codonopsis radix*), paoshen (*Adenophora stricta* Miq.), tutangshen (*Campanumoea javanica* Blume), turenshen (*Talinum paniculatum* (Jacq.) Gaertn.) and hongshen (*Phytolacca americana* L.). Edible plants used for reducing heatiness and relieving summer heat were mainly herbaceous plants, including some vines and trees whose edible parts were the flowers and leaves. Plants whose leaves and flowers are soaked in water for drinking are collectively called “tea,” such as KuDing tea, Tian tea and JinYinHua tea. Apart from general nourishing effects, a few edible wild plants also have some special nourishing effects. Generally, nourishing foods are used to make stews or infused wine with chicken, pork ribs and pig trotters, such as stewed chicken with DangShen and stewed pork ribs with paoshen. Special nourishing plants include Yang-tonifying plants (to improve male sexual function) and brain-nourishing plants. Generally, Yang-tonifying plants are used to make infused wine and drunk, such as Jinyingzi and Yinyanghuo, and brain-nourishing plants are mostly seeds and kernels, such as Hetao. Heatiness-reducing edible wild plants are mostly eaten cold or drunk as tea, such as Ma Lan, Pugongying, and Jinyingzi.

### Quantitative evaluation of Gelao edible wild plants in northern Guizhou

**Fig. 6:**
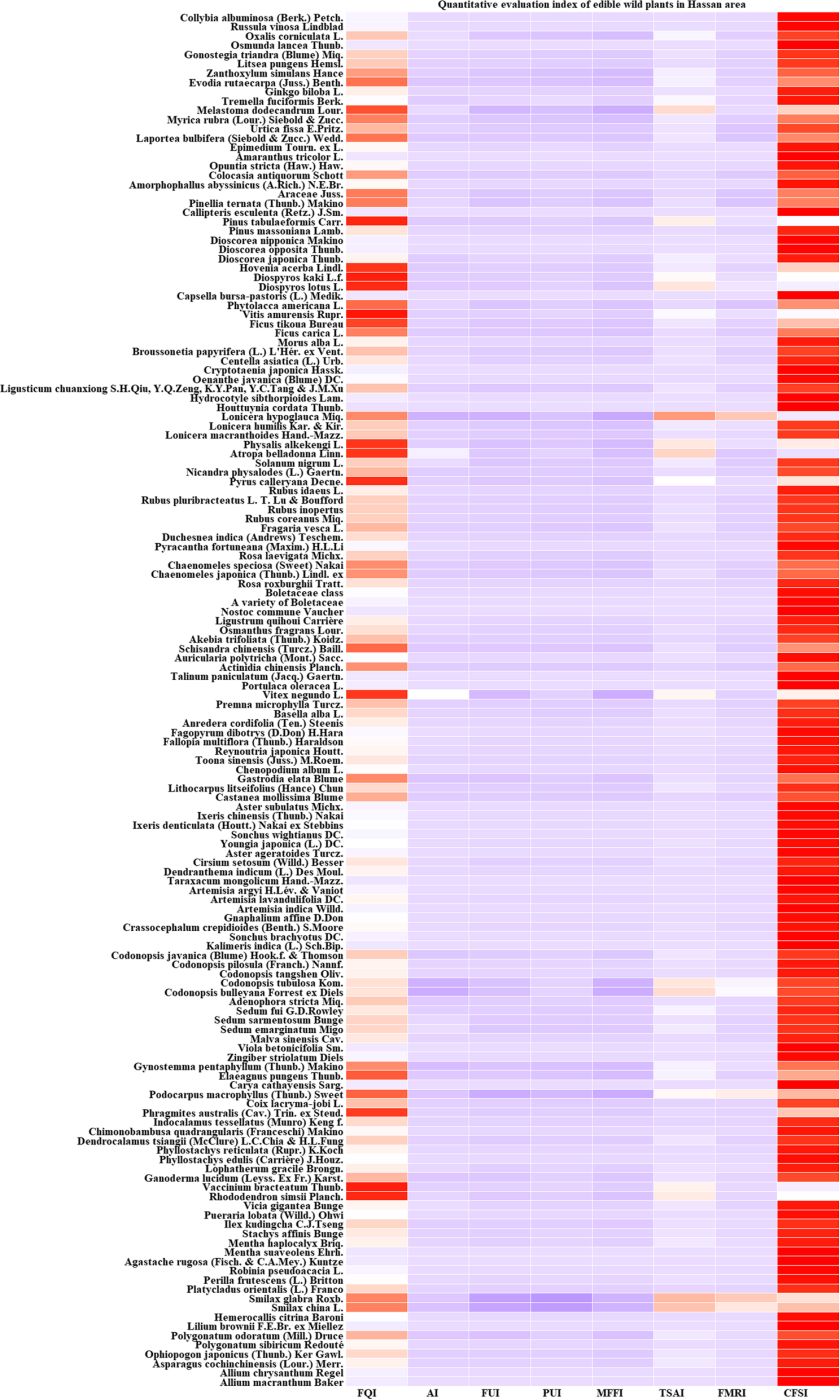
Heatmap of edible wild plants in the Hassan area

The comparison results of the cultural food significance index (CFSI) of Gelao edible wild plants in northern Guizhou are shown in Table 3 and Fig. 6. The edible wild plants in this area were clustered based on the CFSI, and those with broad application and high value, which played an important role in the local people's traditional diet, are highlighted. There were 38 species of plants ranked in the first most important category (CFSI > 500), represented by *H. cordata* Thunb., *M. suaveolens* Ehrh. (Yuxiangcai) (Fig. 4), *Taraxacum mongolicum* Hand.-Mazz., *Callipteris esculenta* (Retz.) J.Sm., *Perilla frutescens* (L.) Britton and *Capsella bursa-pastoris* (L.) Medik. These edible wild plants play an important role in the lives of local people and are the best products on the local people's daily table. These plants are widely distributed in this area and are found almost everywhere. *H. cordata* Thunb., *M. suaveolens* Ehrh., *T. mongolicum* Hand.-Mazz., *P. frutescens* (L.) Britton, *T. paniculatum* (Jacq.) Gaertn., *Agastache rugosa* Kuntze and other plants are the favorite garden plants of local residents and are common in flower beds, vegetable gardens and even flower pots. There were 55 species of plants ranked in the second most important category (500 > CFSI ≥ 100). Fruits (snacks) and vegetables were the main plants in this category, and the CFSI value of wild vegetables was higher than that of fruits, ranking at the top of the second category. These plants are also widely distributed in this area and provide a variety of fruits and vegetables for local residents. The reason for their relatively low CFSI value is mainly related to their edible parts, taste and degree of domestication and cultivation by local residents. There were 44 species of plants ranked in the third most important category (100 > CFSI ≥ 10). Roots (rhizomes) and fruits were the main edible parts of the plants in this category. Moreover, there were many plants with medicinal functions in this category, such as asparagus and *Asparagus cochinchinensis* (Lour.) Merr., *Ophiopogon japonicus* (Thunb.) Ker Gawl., *Mentha haplocalyx* Briq., *Ganoderma lucidum* (Leyss. Ex Fr.) Karst., *C. speciosa* (Sweet) Nakai, *Lonicera macrantha* Spreng. and *P. odoratum* (Mill.) Druce. The fourth most important category (10 > CFSI) had the lowest number of plants, with 14 species. The plants included in this category were mainly plants with special distribution areas, poor taste or special uses, such as *V. negundo* L.

**Table 3:** Quantitative evaluation index of edible wild plants in Hassan area

Plant name	FQI	AI	FUI	PUI	MFFI	TSAI	FMRI	CFSI
*A. macranthum*	47	3	3	4	1.5	10	3	761.40
*A. chrysanthum*	58	3	3	3	1.5	10	3	704.70
*A. cochinchinensis*	22	2	2	3	0.75	9	5	89.10
*O. japonicus*	31	2	2	2	0.75	9	5	83.70
*P. sibiricum*	52	3	2	3	0.75	7.5	5	263.25
*P. odoratum*	28	2	1	3	0.75	7.5	5	47.25
*L. brownii*	49	4	2	3	1.5	9	5	793.80
*H. citrina*	66	4	3	1.5	1.5	6.5	4	463.32
*S. china*	9	2	1	0.75	1.5	6.5	5	6.58
*S. altissima*	7	2	1	0.75	1.5	5.5	5	4.33
*P. orientalis*	121	5	1	2	0.75	7.5	5	340.31
*P. frutescens*	127	4	3	3	1.5	9	5	3086.10
*P. frutescens*	35	3	1	3	1.5	9	5	212.63
*R. pseudoacacia*	88	5	2	1.5	1.5	9	5	891.00
*A. rugosa*	92	5	3	3	1	10	5	2070.00
*M. suaveolens*	136	5	4	3	1	10	5	4080.00
*M. haplocalyx*	22	3	1	3	1	9	5	89.10
*S. affinis*	36	2	1	3	1.5	10	4	129.60
*I. kudingcha*	28	2	2	2	1	6.5	5	72.80
*P. lobata*	54	5	1	3	1	9	5	364.50
*V. gigantea*	19	5	2	2	1.5	7.5	2	85.50
*R. simsii*	24	2	1	1.5	0.5	7.5	2	5.40
*V. bracteatum*	36	1	1	1.5	0.5	9	2	4.86
*G. lucidum*	45	1	1	4	1	9	5	81.00
*L. gracile*	22	4	1	3	1	6.5	5	85.80
*P. edulis*	92	3	3	2	1.5	9	3	670.68
*P. bambusoides f. lacrima-deae*	78	3	2	2	1.5	9	3	379.08
*D. tsiangii*	36	3	1	2	1.5	9	3	87.48
*C. quadrangularis*	73	3	2	2	1.5	10	3	394.20
*I. tessellatus*	135	4	2	1.5	0.75	6.5	5	394.88
*P. australis*	75	3	1	1.5	0.75	4.5	3	34.17
*C. lacryma-jobi*	63	3	2	1.5	1	4.5	5	127.58
*T. wallichiana* var. *chinensis*	11	2	1	1.5	1	4.5	5	7.43
*C. cathayensis*	114	4	4	1.5	1.5	10	5	2052.00
*E. pungens*	34	2	2	1.5	0.5	7.5	3	22.95
*G. pentaphyllum*	27	1	2	1.5	1	7.5	5	30.38
*Z. striolatum*	69	4	3	2	1.5	9	3	670.68
*V. betonicifolia*	18	5	2	3	1.5	7.5	5	303.75
*M. cathayensis*	73	3	3	1.5	1.5	9	2	266.09
*S. emarginatum*	21	5	1	1.5	1.5	7.5	3	53.16
*S. sarmentosum*	32	5	1	1.5	1.5	7.5	3	81.00
*S. fui*	17	2	2	1.5	1.5	9	4	55.08
*A. stricta*	42	1	2	3	1	7.5	5	94.50
*D. bulleyana*	7	1	2	3	1	7.5	5	15.75
*C. tubulosa*	8	1	2	3	1	7.5	5	18.00
*C. pilosula subsp. tangshen*	52	2	2	3	1	7.5	5	234.00
*C. pilosula*	31	2	2	3	1	7.5	5	139.50
*C. javanica*	26	1	2	3	1	7.5	5	58.50
*A. indicus*	45	5	3	3	1.5	7.5	5	1139.06
*S. brachyotus*	83	5	3	2	1.5	9	3	1008.45
*C. crepidioides*	45	5	2	2	1.5	9	2	243.00
*G. affine*	147	5	4	2	1	9	2	1058.40
*A. indica*	78	5	4	1.5	1	9	4	842.40
*A. lavandulifolia*	92	5	3	1.5	1	7.5	3	465.75
*A. argyi*	56	4	3	1.5	1.5	7.5	5	567.00
*T. sect. Erythrocarpa*	135	5	3	3	1.5	9	5	4100.63
*D. indicum*	77	5	2	1.5	1	6.5	5	375.38
*C. arvense var. integrifolium*	38	5	1	2	1.5	7.5	3	128.25
*A. trinervius subsp. ageratoides*	124	5	3	2	1.5	7.5	3	1255.50
*Y. japonica*	36	4	2	2	1.5	9	3	233.28
*S. wightianus*	111	4	3	2	1.5	9	3	1078.92
*I. polycephala*	102	3	3	2	1.5	9	3	743.58
*I. chinensis*	53	5	2	2	1.5	9	3	429.30
*S. subulatum*	92	5	3	2	1.5	7.5	3	931.50
*C. mollissima*	101	2	3	1.5	0.5	9	4	163.62
*L. litseifolius*	32	2	2	1.5	1	9	5	86.40
*G. elata*	54	1	1	3	1	7.5	5	60.75
*C. album*	41	5	2	2	1.5	6.5	3	239.85
*T. sinensis*	98	2	3	1.5	1.5	9	3	357.21
*R. japonica*	63	4	2	1.5	1.5	9	3	306.18
*P. multiflorus*	48	5	1	2	1.5	7.5	5	270.00
*F. dibotrys*	22	5	3	1.5	1.5	7.5	3	167.06
*A. cordifolia*	47	4	3	1	1.5	7.5	3	190.35
*B. alba*	39	4	3	1	1	7.5	3	105.30
*P. microphylla*	63	3	2	1.5	1	7.5	3	127.58
*V. negundo*	13	4	1	2	0.5	4.5	2	4.68
*P. oleracea*	125	5	2	4	1.5	9	3	2025.00
*T. paniculatum*	35	4	2	4	1.5	9	5	756.00
*A. chinensis*	142	3	2	1.5	0.5	9	3	172.53
*A. polytricha*	46	2	2	4	1.5	10	3	331.20
*S. chinensis*	77	2	2	1.5	0.5	9	3	62.37
*A. trifoliata*	132	3	3	1.5	0.5	10	3	267.30
*O. fragrans*	54	5	2	1.5	0.75	9	3	164.03
*L. quihoui*	57	5	2	1.5	1	5.5	5	235.13
*N. commune*	84	5	3	4	1.5	10	3	2268.00
*B. edulis*	127	2	3	4	1.5	10	3	1371.60
*M. esculenta*	58	2	2	4	1.5	10	3	417.60
*A. elata*	117	5	3	2	1.5	9	3	1421.55
*R. roxburghii*	138	4	4	1.5	0.5	9	3	447.12
*C. japonica*	65	3	2	1.5	0.5	9	3	78.98
*C. speciosa*	65	3	2	1.5	0.5	6.5	4	76.05
*R. laevigata*	43	4	2	1.5	0.75	5.5	5	106.43
*P. fortuneana*	117	5	4	1.5	1	7.5	4	1053.00
*D. filipendula*	92	5	3	1.5	0.5	9	3	279.45
*F. vesca*	47	3	3	1.5	0.5	9	3	85.66
*R. coreanus*	58	4	3	1.5	0.5	9	3	140.94
R. inopertus	49	4	3	1.5	0.5	9	3	119.07
*R. pluribracteatus*	66	4	3	1.5	0.5	9	3	160.38
*R. parvifolius*	73	4	3	1.5	0.5	9	3	177.39
*R. idaeus*	57	4	3	1.5	0.5	9	5	230.85
*C. xerophila*	32	2	1	1.5	0.5	7.5	3	10.80
*N. physalodes*	72	2	2	1.5	1	10	3	129.60
*S. nigrum*	23	5	1	2	1.5	7.5	2	51.75
*A. bella-donna*	15	3	1	1.5	0.5	6.5	1	2.19
*A. officinarum*	21	2	1	1.5	0.5	7.5	3	7.09
*L. macrantha*	32	3	2	1.5	0.75	6.5	5	70.20
*L. humilis*	19	3	2	1.5	0.75	6.5	5	41.68
*L. hypoglauca*	7	1	1	1.5	0.75	6.5	5	2.56
*H. cordata*	149	5	5	4	1.5	7.5	5	8381.25
*H. sibthorpioides*	37	5	2	4	1.5	9	3	599.40
*L. sinense* var*. hupehense*	32	3	1	2	1.5	7.5	3	64.80
*O. javanica*	104	4	3	2	1.5	7.5	3	842.40
*C. japonica*	95	5	4	2	1.5	7.5	3	1282.50
*C. inclusum*	31	5	1	2	1.5	7.5	3	104.63
*B. papyrifera*	51	5	2	1.5	0.5	9	3	103.28
*M. alba*	77	4	3	1.5	0.5	10	5	346.50
*F. carica*	68	3	2	1.5	0.5	7.5	3	68.85
*F. tikoua*	59	3	1	1.5	0.5	7.5	3	29.87
*V. amurensis*	44	1	1	1.5	0.5	7.5	3	7.43
*P. americana*	57	5	1	3	1	5.5	1	47.03
*C. bursa-pastoris*	94	5	4	4	1.5	9	3	3045.60
*D. lotus*	22	1	1	1.5	0.5	7.5	3	3.71
*D. kaki*	41	1	1	1.5	0.5	9	3	8.30
*H. acerba*	79	1	2	1.5	0.5	9	3	32.00
*D. japonica*	17	1	3	3	1	9	5	68.85
*D. nummularia*	43	3	3	3	1	9	5	522.45
*D. nipponica*	53	3	3	3	1	9	5	643.95
*P. massoniana*	76	5	3	1.5	0.5	10	3	256.50
*P. tabuliformis*	32	1	1	1.5	0.5	9	3	6.48
*C. esculenta*	139	5	4	3	1.5	10	3	3753.00
*P. ternata*	46	5	1	3	1	6.5	1	44.85
*A. variabilis*	72	5	1	3	1	6.5	1	70.20
*A. konjac*	94	4	2	3	1	7.5	3	507.60
*C. antiquorum*	83	3	2	3	1	7.5	1	112.05
*O. dillenii*	68	2	2	4	1.5	7.5	3	367.20
*A. tricolor*	45	4	4	4	1.5	9	3	1166.40
*E. borealiguizhouense*	25	3	2	4	0.75	5.5	5	123.75
*L. bulbifera*	45	2	2	2	1.5	7.5	1	40.50
*U. fissa*	34	4	2	2	1.5	7.5	1	61.20
*G. hirta*	42	4	3	1.5	0.5	9	3	102.06
*M. rubra*	39	3	2	1.5	0.5	7.5	3	39.49
*M. dodecandrum*	16	3	1	1.5	0.5	7.5	3	8.10
*T. fuciformis*	11	1	3	4	1.5	10	3	59.40
*G. biloba*	34	3	2	1.5	1	9	5	137.70
*T. ruticarpum*	27	2	2	1.5	0.75	6.5	3	23.69
*Z. simulans*	22	3	2	1.5	0.75	6.5	3	28.96
*L. pungens*	53	3	3	1.5	0.75	7.5	3	120.74
*O. lancea*	37	5	4	3	1.5	9	3	899.10
*O. corniculata*	21	5	2	1.5	0.5	9	3	42.53
*R. vinosa*	47	2	3	4	1.5	9	3	456.84
*C. albuminosa*	73	2	3	4	1.5	9	3	709.56

## Discussion

Guizhou Province, located in southwest China, has abundant rainfall and changeable terrain. The special geographical environment has created a suitable environment for plants, and a wide variety of plant resources have also provided abundant food resources to local residents [10]. The results show that, compared with our previous research results on edible wild plant resources in arid areas of northwest China's Loess Plateau (Hassan area), the edible wild plant resources collected in the concentrated areas of the Gelao people in northern Guizhou are much richer in species, edible categories and consumption modes. The Gelao people have rich traditional knowledge of plant identification, medicinal uses and resource protection.

### Gelao people’s botanical understanding of edible wild plant resources

Based on long-term experience, the local Gelao people have accumulated a wealth of traditional knowledge on the rich and varied local edible wild plant resources, not only in terms of their use as food but also as medicine. However, regarding the strict classification of plants, the local residents' level of understanding is limited. For some plants with related species, the local residents often collectively call them the name of their edible parts. For example, many plants in the Caprifoliaceae are consumed as honeysuckle, and individuals can only distinguish them based on leaf size, flower length and color. Only the fruit color (red or yellow) can be distinguished among different *P. fortuneana* (Maxim.) H.L. Li varieties, and the differences among other species are attributed to their differences in light, water and soil nutrient requirements in the growing environment. The tender seedlings of various ferns are collectively called juecai/juetai moss. Some individuals can tell the differences among these ferns, but they are mostly distinguished based on the picking season, taste and so on.

However, not all related plants are treated as the same kind. Although the local residents collectively refer to the fruits of *Rubus* L. (Rosaceae) as paoer/peier, they have named them different types of “bubble” according to their color, taste and picking season; for example, yellow bubbles and black bubbles are named after their color, and sour bubbles are named after their taste. *M. haplocalyx* Briq. is a traditional Chinese medicine [23], and *M. suaveolens* Ehrh. is a close relative that is often used as a fake substitute in traditional Chinese medicine. However, local residents believe that *M. suaveolens* Ehrh. is the genuine *M. haplocalyx* Briq., that is, fish coriander, and that *M. haplocalyx* Briq. is used as the substitute. *Pseudocydonia* is also divided into two kinds by local residents according to the shape of the fruit: one is elongated and medicinal, whereas the other is round and edible.

### Gelao people’s understanding of the medicinal uses of edible wild plant resources

The Gelao people’s understanding of the medicinal function of plants in this area is mainly based on their knowledge of traditional Chinese medicine. Nourishing, heatiness-reducing, appetizing and dampness-eliminating are all descriptions of the efficacy of traditional Chinese medicine [24]. The Gelao people’s description of a plant’s specific medicinal function is also consistent with those of traditional Chinese medicine, but it is relatively much simpler. The heatiness-reducing plants eaten by the Gelao people are generally aimed at inflammatory fever (excessive internal heat) diseases, such as mouth ulcer, gingival inflammation, halitosis, etc., and can also be used to regulate the similar internal heat effects caused by eating spicy hot pot, while plants that can relieve summer heat are mainly used to deal with hot summer weather and prevent heatstroke. In addition, some knowledge comes directly from traditional Chinese medicine or modern medicine. For example, plants whose edible parts are seeds (kernels) are generally considered to have nourishing effects, and the information that *Carya cathayensis* Sarg. kernels can nourish the brain comes from traditional Chinese medicine [25]. This may be directly related to the fact that *T. chinensis* (Pilg.) Florin (HongDouShan) contains taxiresinol, a prominent anticancer drug [26]. However, we do not know whether the fruit of *T. chinensis* can cure cancer.

Gelao people’s knowledge of plant medicinal uses may also be related to the local climate. The region is rich in plant resources that are used as raw materials for fermented foods. These fermented foods include fruit wines, vinegar, sauces, fermented bean curd and fermented beverages. However, due to the influence of industrialization, it is difficult to find Gelao residents who can provide accurate information about fermented foods at present, but we have learned much such information from local supermarkets. In addition, local residents also like to soak fruits in low-alcohol liquor to make fruit wines with various flavors and colors, such as YangMei wine, CiLi wine and FuPenZi wine. In addition to fruit wine, residents in this area also enjoy other types of infused wines. Various plants are soaked in various kinds of wines according to their efficacy in treating diseases, medicinal functions or other special properties. Typical infused wines can be used for dispelling wind and dampness (*Gastrodia elata* Blume), improving male sexual function (*Epimedium borealiguizhouense* S. Z. He & Y. K. Yang, *R. laevigata* Michx.) and to fight cancer (HongDouShan fruit). In most cases, many plant species are mixed and soaked together, and some medicinal wines are soaked with animal medicinal materials.

Some plants that are considered poisonous by modern knowledge are also fully utilized as food by residents of northern Guizhou, the most important of which is the Araceae [27]. Many species of Araceae are called YeMoYu by locals. *Solanum tuberosum* L. and other Araceae are usually steamed and cooked with potatoes, sweet *S. tuberosum* L., and Dioscorea, and there is no information about poisonings from the consumption of these plants. This is somewhat similar to eating *Aconitum* L. plants in some areas of Yunnan [28]. The toxic components in the roots of these plants may be destroyed during high-temperature cooking [29, 30], making the food safe. However, local people also have the habit of eating fresh DuJuanHua. However, we did not obtain any useful information during the study on how they distinguish toxic from nontoxic DuJuanHua. However, information about poisonous mushrooms was mentioned repeatedly, which may be related to the local government's vigorous awareness campaign on the subject. For example, some young children have been taught to sing the following song about poisonous mushrooms: “Red umbrella, white stalk, after eating you’ll be dead…”.

### Gelao people’s understanding of resource protection

Among the edible wild plants with CFSI > 500, except *Osmunda lancea* Thunb., *A. elata* (Miq.) Seem. and *C. bursa-pastoris* (L.) Medik., which are rich in resources and are not damaged by eating (young seedlings and leaves are the main edible parts), a large number of plants are cultivated in the courtyards of local residents, such as *H. cordata* Thunb., *M. suaveolens* Ehrh., *Zingiber striolatum* Diels., *Lilium brownii* F.E.Br. ex Miellez. and *Polygonatum sibiricum* Delar. ex Redoute. The main purpose of cultivation is to facilitate eating, but it is also an effective protection strategy for these frequently eaten resources.

Local residents also consciously protect some rare plants. For example, the whole plant of *G. elata* Blume is not dug, and a certain number of provenances will be reserved so that this valuable medicinal and edible plant resource can sustainably provide food for residents. The collection of *E. borealiguizhouense* S. Z. He & Y. K. Yang has gradually changed from the previous whole-plant digging to the method of collecting leaves and keeping roots. For plants whose roots are eaten, residents basically follow the principle of picking large ones and keeping small ones. At the same time, they will consciously spread the seeds of rare plants to help their population expand, such as *Codonopsis radix*, *T. paniculatum* (Jacq.) Gaertn., and *L. brownii* F.E.Br. ex Miellez. (pearl bud).

Through combining 23 reports which have been published, it is found that the research areas are mainly in Guizhou, Yunnan, Inner mongolia, Gansu, Fujian, Sichuan Province and Tibet. There are 1,912 kinds of edible ethnic plants in these places. Compared with the 151 kinds of wild edible plants collected in Gelao area in northern Guizhou, we have investigated 66 kinds of wild edible plants that have never been published before, such as *Youngia japonica* (L.) DC., *Symphyotrichum subulatum* (Michx.) G.L.Nesom, *Rubus idaeus* L*.*, *Rubus coreanus* Miq., *Nicandra physalodes* (L.) Gaertn. and *Indocalamus tessellatus* (Munro) Keng f., which are mostly local plants of Gelao nationality.

### Current status of the Gelao people’s traditional cultural knowledge

Although the informants were mainly Gelao people, we found that there was no considerable difference between the residents belonging to this ethnic group, their Miao and Tujia neighbors, and the local Han people. This differs from the ethnic groups in northern China, such as Tibetans and Mongolians, who have their own characteristics [31, 32]. The traditional cultural knowledge of the Gelao people is basically only displayed in festivals or performances for travelers. Their knowledge of the uses of wild plant resources is no different from that of the local Han people. The 151 species of edible plants cited by the Gelao informants are found in various recipes or other works by the Han people [10, 33]. The ethnic characteristics of the Gelao people have thus basically died out. At the same time, we also found that the amount of information provided by the informants was positively correlated with age. Most young people under the age of 25 only know that there are certain plants that can be eaten, and they have eaten them before, but they know little about the plants and how to prepare them. In 2020, China has lifted the whole people out of poverty and completely solved the food problem of China Chinese people. Wild edible plant resources of ethnic minorities are mainly used as wild vegetables, condiments or tonics, and only a few varieties are gradually domesticated into daily edible vegetables and become supplementary resources to the existing food resources. But at present, the vast majority of them are only inherited as a traditional culture.

## Conclusions

The Gelao people are a special ethnic group living in mountainous areas of northern Guizhou who is affected by a mountainous geographical environment and a shortage of land resources. Their ancestors had the habit of collecting wild plants as food supplements. During the long-term collection and utilization of wild plant resources, the Gelao people have amassed a great deal of traditional knowledge, which has been passed down and accumulated from generation to generation. However, with the development of the social economy, the traditional knowledge passed down from older generations has been gradually forgotten by the younger generations, and its inheritance is faced with great risks. Through ethnobotanical research, we collect, sort and spread this precious traditional knowledge, which is of great value to its protection. The inheritance of the traditional knowledge on plants is as valuable as that of the traditional skills of ethnic groups with unique characteristics varying among countries and regions [34].

## Data Availability

All data, materials and information are collected from the study sites.

## Abbreviations

5W + 1H: Why What Where When Who How
FQI: Frequency of quotation index
AI: Availability index
FUI: Frequency of utilization index
PUI: Parts used index
MFFI: Multifunctional food use index
TSAI: Taste score appreciation index
FMRI: Food medicinal role index
CFSI: Cultural food significance index
